# Students' social networks are diverse, dynamic and deliberate when transitioning to clinical training

**DOI:** 10.1111/medu.14382

**Published:** 2020-10-13

**Authors:** Anique E. N. Atherley, Laura Nimmon, Pim W. Teunissen, Diana Dolmans, Iman Hegazi, Wendy Hu

**Affiliations:** ^1^ Faculty of Health, Medicine and Life Sciences School of Health Professions Education (SHE) Maastricht University Maastricht The Netherlands; ^2^ School of Medicine University of Western Sydney Penrith NSW Australia; ^3^ Faculty of Medicine Centre for Health Education Scholarship (CHES) University of British Columbia Vancouver BC Canada; ^4^ Department of Obstetrics and Gynaecology VU University Medical Centre Amsterdam The Netherlands

## Abstract

**Context:**

Transitions in medical education are dynamic, emotional and complex yet, unavoidable. Relationships matter, especially in times of transition. Using qualitative, social network research methods, we explored social relationships and social support as medical students transitioned from pre‐clinical to clinical training.

**Methods:**

Eight medical students completed a social network map during a semi‐structured interview within two weeks of beginning their clinical clerkships (T_0_) and then again four months later (T_1_). They indicated meaningful interactions that influenced their transition from pre‐clinical to clinical training and discussed how these relationshipsimpacted their transition. We conducted mixed‐methods analysis on this data.

**Results:**

At T_0_, eight participants described the influence of 128 people in their social support networks; this marginally increased to 134 at T_1_. People from within and beyond the clinical space made up participants’ social networks. As new relationships were created (eg with peers and doctors), old relationships were kept (eg with doctors and family) or dissolved over time (eg with near‐peers and nurses). Participants deliberately created, kept or dissolved relationships over time dependent on whether they provided emotional support (eg they could trust them) or instrumental support (eg they provided academic guidance).

**Conclusions:**

This is the first social networks analysis paper to explore social networks in transitioning students in medicine. We found that undergraduate medical students’ social support networks were diverse, dynamic and deliberate as they transitioned to clerkships. Participants created and kept relationships with those they trusted and who provided emotional or instrumental support and dissolved relationships that did not provide these functions.

## Abstract 2

It is well‐established that relationships are crucial to effective education. Here Atherley et al. explore how relationships change as students transition into clinical training.

## 1 INTRODUCTION

Transitions in medical education are dynamic,^1^ emotional and complex^2^ yet, unavoidable. Transitions are imposed by health care systems (eg changing medical teams/firms) and training programmes (eg from pre‐clinical to clinical training).^1^, ^3^, ^4^, ^5^ Few studies explicitly focus on social and developmental aspects to transitions even though medical educators are increasingly fostering competencies such as collaboration and reflection. In this paper, we contribute to the conversation surrounding social aspects of transitions as we unpack relationships influencing undergraduate medical students transitioning from pre‐clinical to clinical training.

Transitions are dynamic periods^1^ requiring the transfer of previous training (eg from pre‐clinical classroom‐based learning) to the workplace (eg to the clinical environment).^6^ Discourse in transition literature focuses on the associated distress^3^ and anxiety during transitions which may lead to self‐doubt.^7^ Persistent self‐doubt can result in limited speaking up behaviour which is linked to increased medical errors.^8^ However, transitions also offer an opportunity for personal and professional development.^1^, ^3^, ^9^ Thus, entering a new environment can also be a ‘learning asset’^10^, ^11^; the ‘struggles’ experienced when transitioning to being a doctor for the first time can be motivating when supported.^12^ Numerous interventions have been developed to address the threats inherent in transitions within medical education. These primarily focus on bridging the educational gap of knowledge and skills that newcomers experience as they move to new environments.^3^ Although these interventions may increase confidence,^13^ students must develop meaningful relationships and integrate into clinical environments to be able to access opportunities to learn, practice and showcase taught knowledge and skills.^3^, ^4^, ^14^, ^15^, ^16^, ^17^

The social perspective to transitions is important as ‘relationships matter’,^18^ especially in times of transition’.^6^ Social integration is notoriously difficult in clinical clerkships, especially those that are rotation‐based.^1^, ^4^, ^5^ Social integration buffers stress during intense periods^19^ and is one form of social support.^20^, ^21^ Social support is a ‘network‐based phenomenon’^22^ described as any social transaction that may be helpful for the receiver in a particular situation.^6^ Therefore, creating and keeping supportive social relationships is crucial during transitions. There is a gap in the transitions literature calling for more research from a social perspective.^3^ We are yet to know how social relationships influence students’ transition as they leave the classroom and go to a clinical training environment.^3^, ^23^, ^24^ Social network research methods could help us fill this gap.

Social network theory tells us that behaviour and performance are the result of the way individuals are tied to their social connections.^25^ Social network analysis (SNA) has been invaluable in understanding relationships in undergraduate medical education. We know now that medical students choose friends of similar sex and background during medical training,^26^ that undergraduate students’ relationships predict their performance,^27^ that institutional allocation influences friendship development^26^ and even that faculty's social networks influence learning about teaching.^28^ However, social networks have not yet been explored in relation to transitions in medical training, including how they influence students’ transition experiences. In recognising that learning is a collaborative process, we anticipated that exposing the social fabric of relationships between students and others would enable us to consider how social structures and individual preferences interact during the transition to clinical training.

We therefore used SNA to examine the social interactions of undergraduate students, studying their relationships and connections^29^ during their transition to the clinical environment. Specifically, we sought to answer the following research questions:

**RQ1:** Who are in the social networks of undergraduate students transitioning to the clinical environment?

**RQ2:** To what extent do undergraduate students’ networks change as they transition to the clinical environment? What are the mechanisms behind these changes?

## 2 METHODOLOGY

### 2.1 Context

This study took place in the five‐year medical programme of Western Sydney University (WSU), Australia. Every year, approximately 130 students (primarily school‐leavers) enter the medical programme at WSU. The programme comprises two pre‐clinical years with a PBL curriculum and three clinical years with rotation‐based clerkships (also called attachments or placements). We took our sample from an ethnically diverse sampling frame at WSU.

### 2.2 Researchers’ stance

We conducted this study within a social constructivist paradigm; we believe that individuals create meaning through their interactions with others.^30^ In addition to concepts from social network theory mentioned above, we were further sensitised by theoretical constructs from landscapes of practice^10^—an iteration of community of practice theory.^31^ This was a useful conceptual tool because, in medical education, new clinical students interface with boundaries surrounding communities of *clinical* practice (CoCPs)^32^ as they attempt to socially integrate when transitioning in and out of numerous undergraduate clerkships in the landscape of clinical practice. Likewise, social network research considers the location of the boundary around a network of people who are interacting. Landscapes of practice complements social network analysis given the social perspective to learning and overlapping concepts such as *brokers* and *boundaries*.

### 2.3 Participants

We collected network data from a convenience sample of eight undergraduate medical students. Our eight participants included four males and four females between the ages of 19 and 24. Participants identified their nationality/ethnicity as being: Chinese, Caucasian, Malaysian, Indian, Bangali, Sri Lankan and Middle Eastern. We will refer to the participants as David, Kenneth, Greg, Nicole, Sonita, Elizabeth, Tyrell and Kendi. David and Kendi had some psychological distress during the data collection period due to issues related to training. However, this emotional distress was promptly addressed as both students went to the institution's counsellor and David took two mental health days as allowed.

### 2.4 Procedures

To collect data for the current study and answer our research questions, we used an egocentric approach to SNA by focusing on specific individuals instead of a complete network approach.^33^ We therefore focused on the social networks of individuals (*ego*) made up of people called *alters* whose relationship is called a *tie*. We collected data using interviews at two time‐points, five months apart, allowing us not only to understand network structure, but also network change and mechanisms functioning within networks. The first interview was within the first two weeks of the first clinical year (T_0_), and the second was four months later (T_1_).

**FIGURE 1:**
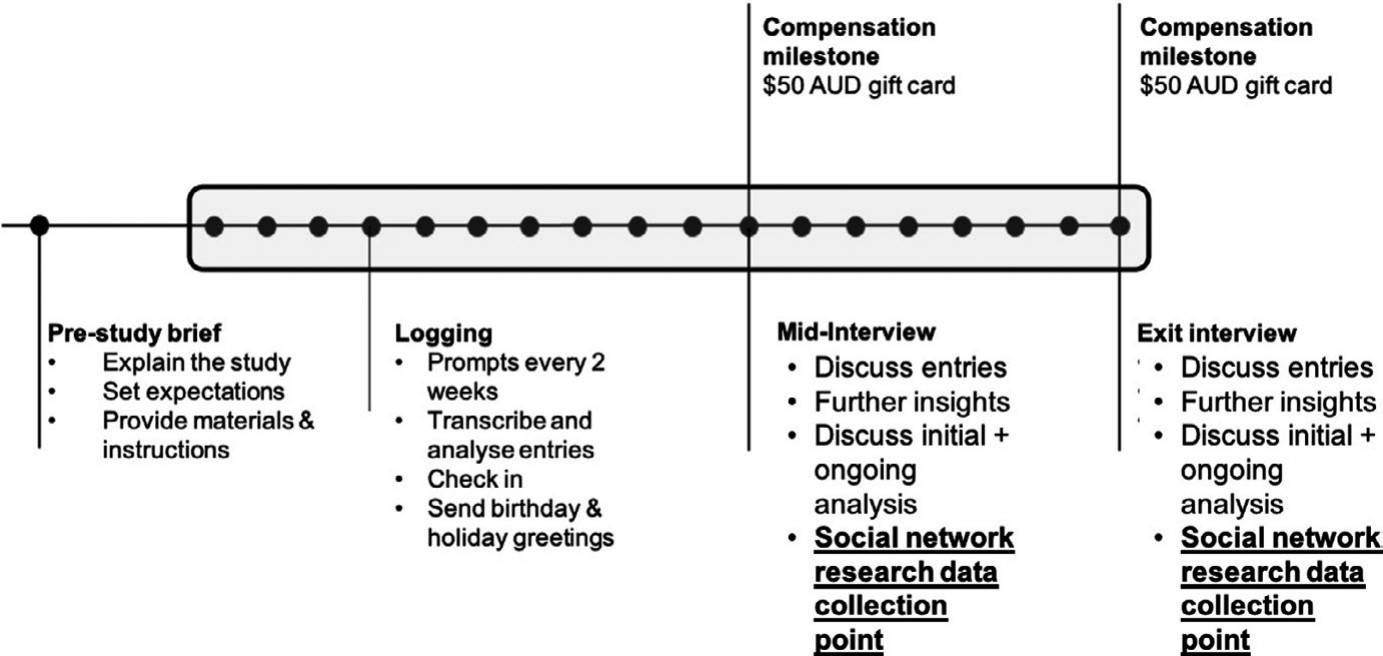
Data collection procedures for two related but distinct studies. Bold and underlined text indicates the data collected for the current study

Our research programme focuses on the transition from pre‐clinical to clinical training, and our early literature review stimulated us to explore a number of distinct, yet related, topics. In a previous narrative inquiry, we investigated how students storied their transition and thus the lived reality they experienced. Nine students regularly completed audio‐recording diaries for nine months, and they then participated in two interviews yielding over 60 data points (See Figure 1). We report on these findings separately.^34^ In the current study, we focused on the specific influence of social relationships when students transition to clinical training. The data collected for the current study were different to that for our previous study, and answers separate research questions. We collected data for the current study at the interviews conducted for the previous study using a separate interview guide as we describe below. We did this for logistical reasons and to respect participants’ time. Eight of the nine students participated in the current study as one did not respond to reminders about the interviews, and thus, this student was not a part of our exploration of social relationships.

**FIGURE 2:**
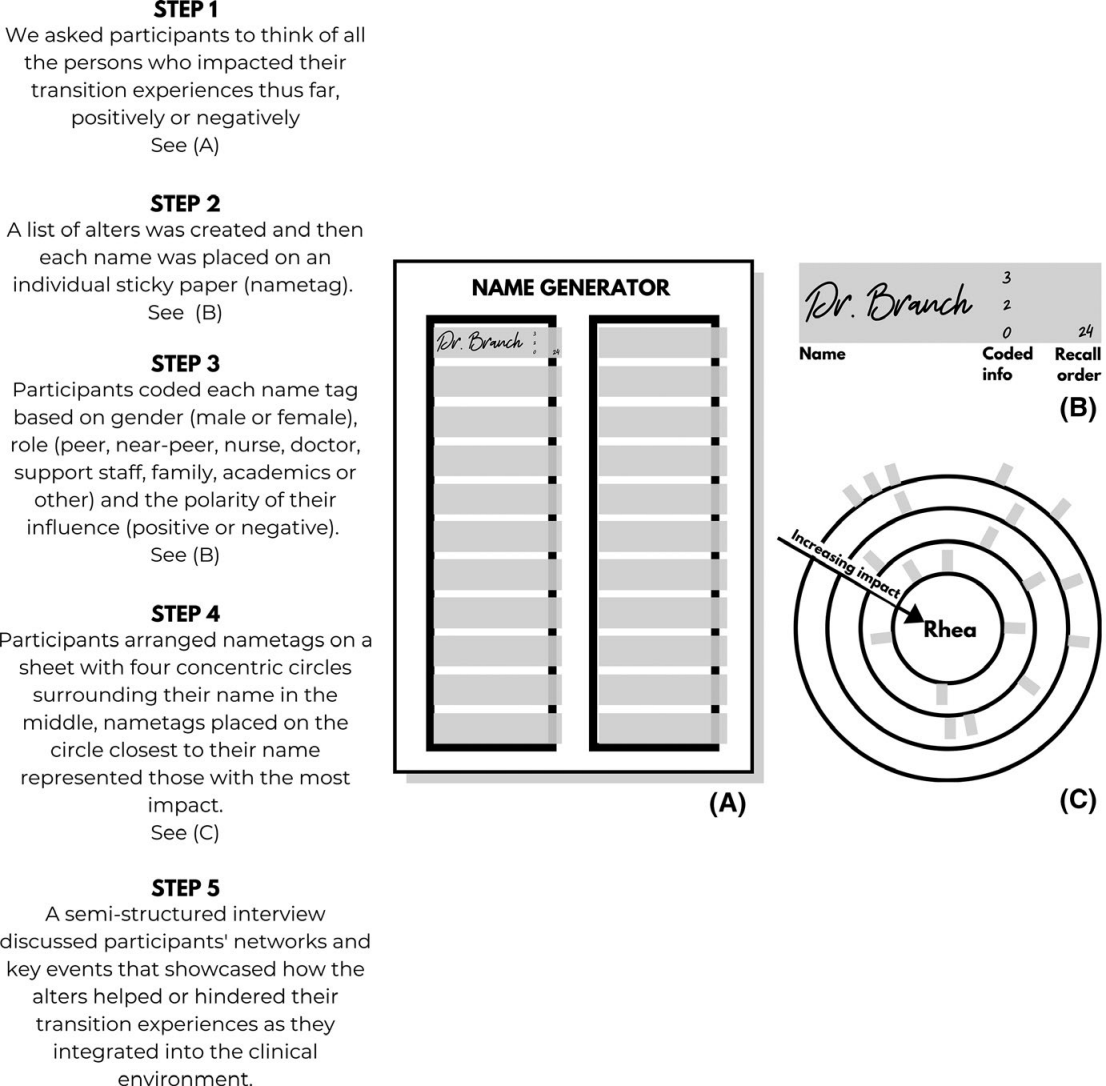
These images show the procedures used in this study to create target sociograms which represented the support networks of our participants. A, shows the name generator template where participants listed all those who influenced their transition experiences on name tags. B, shows a coded, labelled name tag. C, shows how a participant, Rhea arranged her name tags in a way that those who had the greatest impact were on the inner circles and those who had the least impact were on the outermost circles. In this sample, 20 nametags represent 20 alters in Rhea's network

During the study period relative to this paper, participants experienced three to four clerkships each five‐week duration. To capture each participant's social support networks, we facilitated systematic identification and consideration of alter choices, relationships and level of impact.^35^ Explicit methodological details and sample images can be found in Figure 2.

We used qualitative data collection through individual interviews at T_0_ and T_1_ to collect egocentric network data from our eight participants. This allowed us to capture data on mechanisms functioning within the social support networks of participants. AA conducted all interviews following two pilot sessions on persons outside of the sample. No changes in the interview process were made. First, we generated names of alters by having participants complete a list after asking them to: ‘think of all the persons who have impacted your transition experiences thus far, positively or negatively. Write each person down’. (Figure 2A). Each alter's name was written on a small Post‐it™ (nametag). AA coded each nametag to indicate the gender, role and whether each alter had a positive or negative influence (see Figure 2B). Participants were free to add alters at any time during the interview. We did not limit the number of alters that participants could indicate, so as not to compromise data quality.^36^, ^37^ Participants then arranged each nametag on one of four concentric circles surrounding their name (See Figure 2C). The innermost circle represented alters who had the most impact on the participant, and the outermost circle represented those who had the least impact. We also asked participants to indicate relationships between alters with circles and lines to connect them. The final output is called a target sociogram (See Figure 2C). Using a semi‐structured interview guide (Box 1), we discussed network‐level and alter‐level influences including key events that made visible how alters helped or hindered participants’ transition experiences. Four months later, these data collection procedures were repeated (T_1_). Participants reflected and discussed any changes in their networks at T_1_. We did not show participants their previous networks at the second interview to avoid influencing alter choices at T_1_. This qualitative approach to SNA captured narratives that ultimately expressed how and why relationships were created, kept or dissolved.^37^, ^38^

**Semi‐structured interview guide: sample questions**

**Table:** 

When did this relationship develop? Who initiated it?
In what way does this person help or didn't help you to transition to the clinical environment?
What kind of information did they share that impacted your transition experiences?
How are your relationships created/kept/lost?

We used an audio‐recorder and video‐camera to record the interviews, and the workspace participants used to create their target sociograms (participants not in frame). Video‐recording helped us identify which alters participants were referencing as they referred to their sociograms as they spoke. Each interview for our research questions lasted between 45 minutes and 90 minutes depending on participants’ network size. In total, participants were interviewed about their social networks for 93 to 194 minutes on average over the two interviews. All audio‐recordings were transcribed verbatim. AA wrote reflective notes after each session. All target sociograms were recreated using Microsoft PowerPoint®, and we changed all names to pseudonyms. Participants were able to receive copies of their target sociograms and could make changes if necessary; no changes were made.

### 2.5 Data management & analysis

This is a mixed‐methods social network analysis which is defined as ‘any study that draws from both qualitative and quantitative data or uses qualitative and quantitative methods of analysis and thoughtfully integrates the different research strands with each other’ (p. 20).^28^ This afforded us richness and interpretative depth through considering the quantitative and qualitative dimensions of the social support networks of participants in our sample. Firstly, AA created a spreadsheet of all alters based on the coded nametags. We also captured strength of the interaction through the concentric circle placement (using a score of 4 for the innermost circle and 1 for the outermost circle). We compared the composition of roles of all alters in all networks at T_0_ (n = 128) vs T_1_ (n = 134) using Pearson's chi‐square statistic.

All transcripts and target sociograms were imported into Atlas.ti for data management and analysis. AA conducted inductive thematic analysis of interview transcripts, target sociograms and videos. AA, DD, PWT and WH and IH collectively discussed the raw data early in the research process. In addition to these discussions, interpretations were liberally discussed with LN or WH and then the entire research team as insights unfolded. Together, our synchronous face‐to‐face discussions and iterative asynchronous e‐mail exchanges shaped our results. We discussed divergent interpretations in online meetings and through tracked changes and comments on initial drafts of the results section.

### 2.6 Ethical considerations

This study received ethical approval through Western Sydney University (WSU). Due to the intimate and personal nature of this research, we have taken care to ensure anonymity through all stages of research and within this paper. We have not indicated the calendar year of data collection, specific participants’ ethnicity nor specific position titles to protect the identity of our participants and alters. All names in this paper are pseudonyms.

### 2.7 Researcher positioning

We believe the transition to clinical training is a social process through which learning, and development, occurs. Some of the authors are doctors with clinical experience (AA, PT, IH and WH). LN has experience conducting and supervising qualitative social network analysis. PT, DD, IH, WH and LN are established qualitative researchers and experienced medical educators. This international team spans a wide range of geographical contexts (Australia, Barbados, Canada, Egypt and The Netherlands). The multifaceted perspectives of the research team increase the information power in this study.^39^ The research relationship between AA and participants, as described earlier, boosted the quality of their interactions.^39^ AA had no hierarchical relationship with the participants as she was not engaged in any teaching appointments and had no role in their assessment or progression.

## 3 RESULTS

We first describe the network structure in our sample at both T_0_ and T_1_ to gather network‐level (size, diversity) and alter‐level insights (gender, roles, strength of ties). This answers RQ1 and tells us, both quantitatively and qualitatively who are in the social networks of students transitioning to the clinical environment. To answer RQ2 (how do networks change and underlying mechanisms for change), we quantitatively and qualitatively compared the alters at T_0_ with the alters at T_1_ for insight into network change and whether ties were created, kept or dissolved. We also explored whether the composition of networks changed. To answer the sub‐question of RQ2, we qualitatively describe the mechanisms functioning to exert network change further integrating the conclusions given by the previous quantitative analysis.

### 3.1 Network structure

**FIGURE 3:**
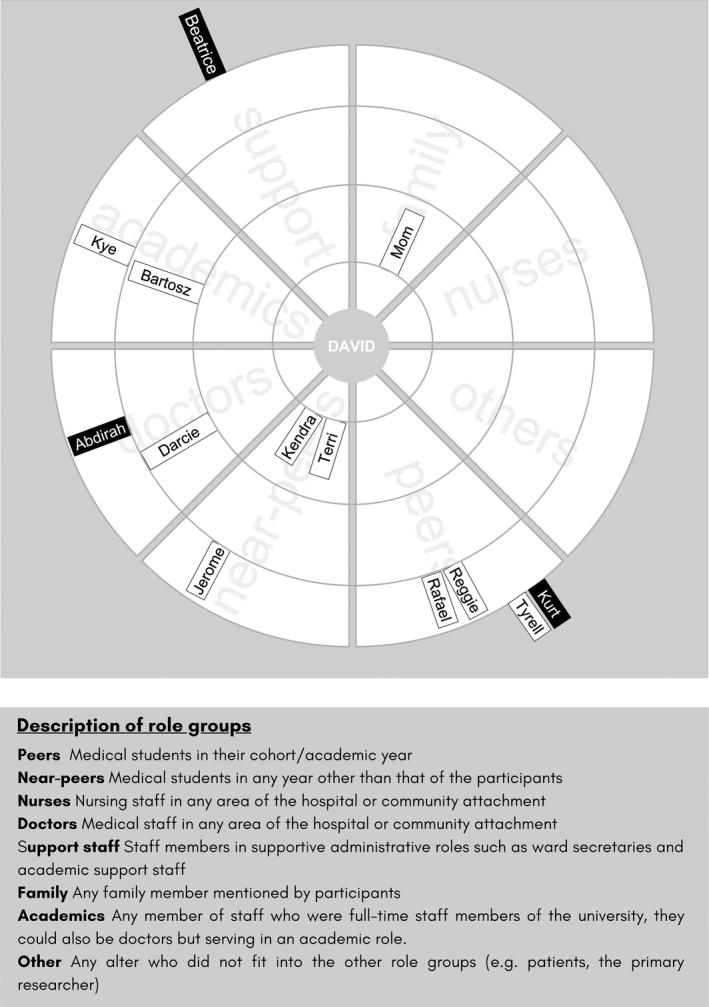
David's network at T_0_ had 15 alters from 6 role groups (see below). This network was made up of a variety of alters including near‐peers, peers, doctors, academics, admin staff and family. His most significant ties were with females—His mom (family), Terri (near‐peer) and Kendra (near‐peer). His least significant ties were with Tyrell (peer) Kurt (peer) and Beatrice (support staff); he had negative, interactions with the latter two

**FIGURE 4:**
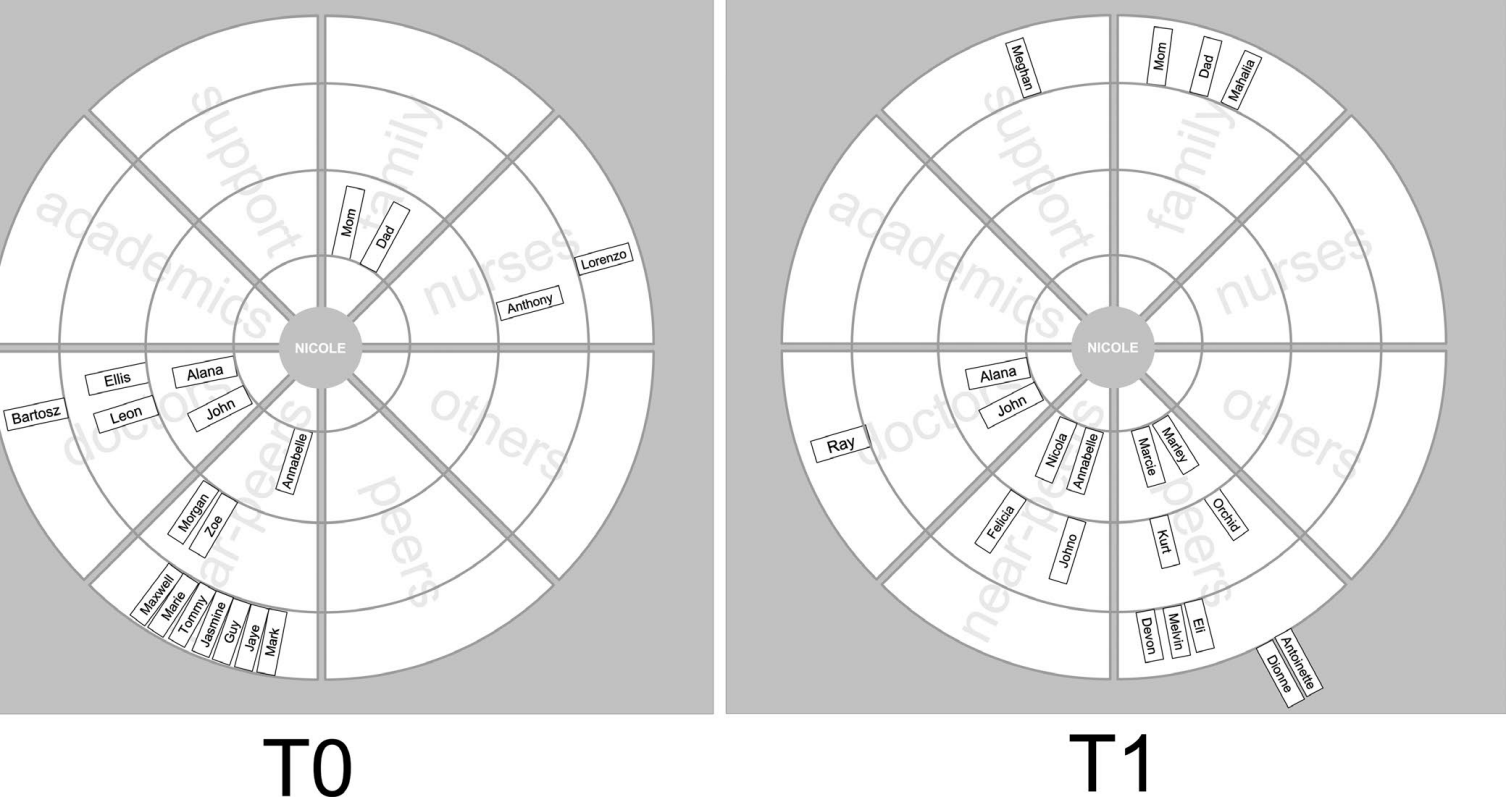
Nicole's network at T_0_ (n = 19) and T_1_ (n = 20). Nicole's initial network included mainly near‐peers and doctors with significant impact from her family. At T_1_, Nicole has developed many relationships with her peers and her bonds with near‐peers have weakened. Her relationships with nurses also diminished though she gained a tie in a support staff member. Relationships with her family members lost significance but remained present

During the initial two weeks of their *first* clinical clerkship, eight students interacted with a total of 128 unique alters (approximately 16 alters per student). Network size ranged from 12 to 22 alters at T_0_. Networks were diverse as is evident on sociograms in Figures 3 and 4. These figures are visualisations of two participants’ networks, Figure 3 shows T_0_ alone for David, and Figure 4 shows T_0_ and T_1_ for Nicole. The figure shows alters on pinwheels so readers can easily visualise which groups the alters in participants’ networks represent (labelled wedge) and also the strength of alters’ influence (strongest influence is indicated closest to the centre and receives a score of 4) and polarity of their influence (black means negative interaction). We have three key findings from T_0_ network data. Firstly, most networks were diverse and comprised mostly of doctors (n = 39; 30.5%), near‐peers (n = 23; 18%) and peers (n = 22 (17.2%); see Table 1, Figures 3&4). Secondly, academics, nurses, support staff and peers were often placed on outer circles as compared to doctors, near‐peers, others and family (see Figures 3&4). Lastly, a few alters appeared in more than one participant's networks. These alters were primarily in institutional positions and included an academic physician and a student‐voted administrative role.

**Table 1:** Proportion of alters in each of 8 roles at T_0_ and T_1_showing increased doctor and peer ties and decreased near‐peer and nursing ties.

Role	T0	T1
	N (%)	N (%)
Doctors	39 (30.5)	53 (39.6)
Family	12 (9.4)	15 (11.2)
Near‐peers	23 (18.0)	8 (6.0)
Peers	22 (17.2)	38 (28.3)
Nurses	11 (8.6)	2 (1.5)
Support staff	2 (1.6)	2 (1.5)
Academics	9 (7.9)	5 (3.7)
Others (patients, mentees, researcher)	10 (7.8)	11 (8.2)

	Value	*df*	*P*
χ^2^	22.6	7	0.002
Likelihood ratio	23.8	7	0.001

### 3.2 Networks on the move

Networks were dynamic and alter composition changed over time. At T_1,_ there were 85 new ties, 99 dissolved ties, and 28 kept ties in the networks of our eight participants, and this led to the total number of ties at T_1_ being 134. Due to the proportions of dissolved ties and kept ties, students’ networks did not significantly get bigger over time; average ties per network went from 16 at T_0_ to 16.75 at T_1_. The overall network change between T_0_ and T_1_ varied between −5 and +15 alters. In Figure 4, we instantly see more peer ties and less near‐peer ties for Nicole; this pattern was seen in most network maps. The role composition of networks was significantly different between time‐points (χ^2^ = 22.6; *P* = 0.002); see Table 1.

#### 3.2.1 Mechanisms for network change

The overarching mechanism for network change was through deliberate network management by our participants. Students intentionally created, kept or dissolved ties depending on emotional or instrumental support factors. Some alters provided both types of support.

#### 3.2.2 Emotional support

Emotional support was often provided by peer roommates, family and sometimes patients. Participants made deliberate decisions to create, keep or dissolve a tie based on an assessment of whether they could trust alters or whether they were physically accessible.

It took many participants time to *trust* their peers with their concerns and questions; this eventually led to the creation of peer ties we see above at T_1_. Both female and male participants revealed that at T_0_, they feared that by admitting to experiencing struggles and knowledge deficits, they would be seen as weak by peers. Some participants did not trust that their peers could help, as their experiences were similar. By T_1_, most participants recognised their peers as an important source of support and learning, and they trusted them more over time. For example, Nicole reflected:

…I think maybe I should have opened up a bit earlier if I had any troubles… to people in my cohort a bit more… I mean I don’t feel like I've had a lot of issues, but I would, kind of, feel a bit embarrassed to bring up issues. I didn’t want to seem weak in front of other people. But now I just tell people [peers] everything… It really helped me not overthink things and to keep things into perspective so I should have just done it earlier. *Nicole, female, 21 years, T1*

Perceived *accessibility* of ties determined whether they were dissolved. For example, near‐peers disappointed some participants who felt that near‐peers had their own education to focus on and could not help them. This likely led to a loss of near‐peer ties that we see in Table 1 and in Figure 4. Below, David expressed his disappointment when Terri did not live up to the expectations he had for her support and friendship.

Terri, I was supposed to see her that day after our meeting, she had other commitments…Terri really made me feel bad because I was really desperate for some help and she left me hanging out to dry…that’s why there is no senior [student] in this picture right now…no one helps… *David, male, 22 years, T_1_*

Trust and perceived accessibility resulted in family ties being stable over time. Family were one group on whom participants always felt they could reliably depend during stressful experiences. Participants welcomed the outside perspective and noted maintaining their family ties was beneficial to their overall well‐being. At both T_0_ and T_1_, family remained in participants’ network, averaging being placed on the second circle from the centre on target sociograms. Below Kendi describes how her dad was reliably supportive resulting in him appearing on her sociogram at both T_0_ and T_1_.

…when I go through, like hardships or obstacles and stuff, um, my Dad’s always there to talk to me if I’m feeling upset and he’ll – because, like, he’s been through – like, he’s not a doctor but he’s been through, like similar stuff, like [a] transition from uni[university] to being, like go to work and then he’d know sometimes it can be hard and he would tell me to, like encourage me… *Kendi, female, 20 years, T_0_*

Contrastingly, David recognised the diminished influence of his mother on his transition, feeling she could not understand as she was from outside the ‘medical world’; that tie was dissolved at T_1_. A few alters had similar thoughts.

In practice, emotional support was often provided mainly through facilitating venting or by alters nurturing participants’ self‐esteem. Venting was an important emotional function that permeated all narratives. It encouraged participants to re‐engage once they off‐loaded and discussed their stresses and concerns. Venting could help shape many participants’ perspective, occurring within relationships that were founded on trust, often with alters on their innermost circles on sociograms (family members, peers and resident doctors). Below, Nicole described how Annabelle facilitated her venting. It is clear that Annabelle was a close, trusted friend by her proximity to the centre at T_0_ and that she persisted at T_1_ in Nicole's network, as seen in Figure 4.

…she [Annabelle] was helping me in that way and say if I was having a bad day, I could just, like, vent to her and she could help me see perspective *Nicole, female, 21 years, T0*

Interestingly, some patients (categorised under the role ‘other’) increased participants’ confidence and validated their presence in the community thereby nurturing their perceived value to the clinical community.

…she [patient] also said, the cannula was good, it was very gentle. So that was good for me. And it felt good that I tried to reassure her a bit. *Tyrell, male, 19 years, T_1_*

Contrastingly, some ties functioned as a source of emotional conflict or increased stress. Some students felt ignored or belittled by persons on their networks. Some ties caused tension and did not neatly fit as a positive or negative interaction. This usually instigated strain on participants’ time, having to choose between their ties and medicine. Interestingly, there were fewer negative ties described at T_1_. Over time, some students were able to use negative interactions to develop personally and professionally:

…even though it wasn’t a great first‐day experience, they made me realise that not everyone will be trying to help me, and I have to make it work by myself. So, I started actively asking questions and trying to, help them as much as I can, and trying to ask questions, trying to help my learning instead of them asking me. *Kendi, female, 20 years, T_1_*

#### 3.2.3 Instrumental support

Instrumental support was often provided by doctors and near‐peers. Participants made deliberate decisions to create, keep or dissolve ties based on an assessment of an alter's expertise or serendipitous interactions with alters who provided academic guidance and shared cultural norms for engaging in a new clerkship environment.

Some participants deliberately sought out the expertise of doctors in particular. Additional doctor ties were created as students experienced more clerkships (see Table 1). Doctor ties were kept if they made participants feel valued, shared their own experiences, recognised participants’ knowledge and skills and showed interest in their development. Below, Nicole notes the influence of Alana, a registrar physician in Nicole's network at T_0_ and T_1_ (see Figure 4). Even though Nicole left Alana's department, five months later, Alana was kept as an alter that Nicole continued to contact for advice.

…it was a big thing for her [Alana] to make sure that everyone was really comfortable…and are we doing okay…you know, she had experiences with, you know, maybe people not being as nice to her…when she was a student, so it was important for her for me not to feel that way… *Nicole, female, 21 years, T_0_*

Students liked when doctors provided opportunities for students to become part of the clinical environment by giving them tasks and roles that integrate them into everyday patient care and sharing their expertise and knowledge with them. This facilitated social integration by cultivating a sense of belonging to the clinical team. Doctors who facilitated such integration persisted in networks over time.

…he trusts me with things; “Go examine and tell me what you find,” okay? Then he’ll type it and that’s it, and it makes me feel more responsible… *David, male, 22 years, T_1_*

When students were not given this opportunity or the environment did not facilitate integration, this left them feeling like they were not yet transitioning. Below, at T_0,_ David struggles with not being invited to participate in clinical tasks.

…he’s my supervisor for GP, I don’t think that he understands that I have to act like a clinician at this point. He treats me as a baby student and I don’t like that… *David, male, 22 years, T_0_*

Guidance was especially important at T_0_ and at T_1_, was increasingly provided by peers contributing to the creation of numerous peer ties. Guidance mainly included sharing advice about cultural norms and tips for working as a clinical student. In the quote below, Sonita discusses the guidance provided by resident doctors during her first week of clerkship.

they [residents] just always asked us questions and then, like, they went through the guide as well, which I don’t think a lot of doctors do…so they were like trying to teach us …at least one topic, over coffee …even though they were really busy with their work as well. *Sonita, female, 19 years, T_0_*

Some participants recognised the reciprocal nature of some ties, especially peer relationships. They recounted how helping others helped them help themselves. This support function of helping others was primarily seen at T_1_ as compared to T_0_.

…I do think I influence my cohort…I go to them for advice, they also come to me for advice. I’ve shared my experiences with them as well, so I think that people — my peers later come to me as well. And they do take in whatever I said, and they take that into consideration as well. *Nicole, female, 21 years, T_1_*

## 4 DISCUSSION

This study explored structure, change and mechanisms functioning in the social networks of medical students transitioning from pre‐clinical to clinical training. We found that the social fabric of relationships as students transition from pre‐clinical to clinical training is indeed a complex, dynamic blend. Our study further strengthens the link between social networks and transitions in the literature; two entities previously examined in isolation.^6^ Despite the widespread acceptance of sociocultural theories of learning, including communities or landscapes of practice theory being applied to workplace‐based learning, this study appears to be the first social network analysis informed by qualitative methods that explores social networks as they relate to a transition during the medical education continuum. Through data collection at two time‐points, we are confident in our explanatory power regarding *how* and *why* relationships relevant to medical training are created, kept or dissolved. Our qualitative data collection methods showed us that the social support networks of our sample were *diverse*, *dynamic* and *deliberate*.

Networks were diverse and were not confined to the boundary surrounding clerkship communities or even the landscape of clinical practice.^10^ Using an egocentric approach, we did not assume that networks comprised of people in pre‐defined boundaries such as in CoCPs^32^ and complete network SNA.^33^ Additionally, we expected that students would have a larger support network a few months into clinical training. This did not happen, contrary to teacher networks participating in an instructional development programme.^40^ The rotation‐based clerkship model is likely responsible for some of the failure we saw for support networks to grow due to a lack of continuity.^41^ Lastly, future research could explicitly explore the influence of alters’ ethnicity vs students’ ethnicity.

Networks were dynamic. During the initial weeks, students missed out on potentially meaningful peer interactions due to feelings of competition and not wanting to appear weak. Promoting speaking up and normalising struggles could help minimise stigma.^42^ These findings are in keeping with the proposed challenges of peer groups in the medical education literature.^43^ Supportive peer relations may improve perceived psychological safety and improve a focus on learning instead of impression management.^44^ We found that some family ties reduced in strength just five months into clerkship. This could be a part of the professional inclusivity and simultaneous social exclusivity as students become doctors.^45^ With concerns of diminished work‐life integration contributing to physician burnout^46^ and retrospective realisation of reliance on family and friends to cope,^47^ maintaining supportive family ties should be encouraged. The loss of nurse ties and lack of meaningful ties with other professionals (eg physiotherapy) could counteract interprofessional collaboration competence^48^; this could be explored in future research. The dynamic nature of networks we found in this study relates to ‘tie churn’, a concept in SNA theory which describes the stability and change in networks over time using ratios of new and dissolved ties to all and kept ties.^49^

Networks were curated through deliberate network management. This is in line with other social network research which considers 'network intentionality'.^50^ We were able to not only describe tie churn but also underlying mechanisms behind why ties were created, kept and dissolved over time as students transitioned to the clinical environment. While we expected networks to grow, having a deliberate, small network could be more beneficial during the emotionally complex transition period. Additionally, network intentionality could be the result of becoming aware of one's networks and thus actively seeking out or dissolving existing ties. Our qualitative data found this intentionality to be dependent on the provision or not, of emotional and instrumental support. Our sample intentionally dissolved relationships with alters who did not provide either emotional support (eg unable to trust them or they were not accessible) or instrumental support (eg did not have enough expertise or did not provide academic guidance). Notably, we may have made students aware of their networks during our first data collection and future research could explore whether becoming aware of one's network could relate to network intentionality in learners.

### 4.1 Strengths and weaknesses

Our findings are limited in transferability by our single‐institution design; however, we aimed for thick description of our processes to make institutional generalisability possible. Our participants experienced a varied combination of clerkships during the study period which allows some transferability outside of any particular clerkship. We acknowledge that transferability is limited but we focused on specific clerkship contexts so as to make data interpretation feasible yet allow comparison within our varied and intense dataset. Using egocentric networks provided insight into understanding both mechanisms functioning in clinical students’ social networks over time. Our findings are likely generalisable to the phenomenon of interest and not the sampling frame.^51^ For example, the concept of deliberate network management is likely generalisable to all transitioning medical trainees given it being found not only here but relates to network intentionality in general SNA literature. Eight participants may appear low; however, we were interested in the 128‐134 ties and properties of those ties.^52^ Given the intense data collection methods, only a small number of participants were necessary to produce this rich, multimodal dataset at two time‐points.^39^ We thus believe this dataset contains sufficient information power^39^ to answer our research questions.

During a few interviews, AA’s influence on some participants was clear as she was mentioned in sociograms and transcripts. As mentioned, AA was known to participants as a researcher exploring their transition to clinical training through their participation in a previous study. We recognised AA’s influence on participants might have varied over time. However, following realising the concrete influence AA had on participants in multiple interviews, we discussed the potential negative implications of AA’s research relationship. These effects could include that participants felt pressured into further data collection by participating in this study thus increasing their participant burden. However, we discussed the impact of their participation in the interviews and found that the relationship between AA and participants allowed them to be open in the discussions relevant to this paper. From participants, we learned that we unintentionally conducted a reflective intervention by having participants reflect on their relationship and experiences in both this and the other related study we described earlier. For example, one participant had to take time off for psychological reasons and noted he used the opportunities for reflecting in audio‐diary recordings to work through his issues and he realised that contemplating his social network allowed him to identify who he could lean on during difficult times. Due to the reflective nature of our data collection, we agree with Froehlich and Gegenfurtner that mapping one's network can potentially can be a useful exercise during stressful periods between phases.^6^

### 4.2 SNA and health professions education in the future

Qualitative and mixed‐methods SNA research could benefit health professions education inquiries, as it has general education research.^17^ Network development could be a social outcome of medical training^53^ and could broaden our scope which is dominantly focused on knowledge acquisition and skills training during transitions. SNA could become a marker of social integration through measuring of network size and other measures.^6^ Mapping social networks could help institutions recognise *brokers* ^10^ and support them. More SNA research in other health professions education fields, like allied health,^52^ could provide holistic data on how networks of health professions interact or not.

## 5 CONCLUSIONS

This is the first social networks analysis paper to explore social networks in transitioning students in medicine. We found that undergraduate medical students’ social support networks were diverse, dynamic and deliberate as they transitioned to clerkships. There were more peer and doctor ties and less near‐peer and nurse ties over time. Students’ deliberate decisions to create, keep or dissolve relationships were based on whether alters provided emotional (eg were trustworthy) or instrumental support (eg provided academic guidance).

## CONFLICT OF INTEREST

None.

## AUTHOR CONTRIBUTIONS

AA, LN, DD, PWT, IH and WH conceptualised and designed the study. AA collected the data. AA, LN and WH analysed the data. AA, LN, DD, WH, IH and PWT were involved in data interpretation. AA produced the first draft of the paper, but all authors contributed to iterative drafting and refinement of the manuscript. All authors approved the final version of the manuscript for submission.

## ETHICAL APPROVAL

This study received ethical approval through Western Sydney University.
